# HPV16 E6-Activated OCT4 Promotes Cervical Cancer Progression by Suppressing p53 Expression *via* Co-Repressor NCOR1

**DOI:** 10.3389/fonc.2022.900856

**Published:** 2022-07-07

**Authors:** Shujuan Shu, Zhi Li, Liu Liu, Xia Ying, Yina Zhang, Ting Wang, Xiaoye Zhou, Peiyue Jiang, Weiguo Lv

**Affiliations:** Department of Obstetrics and Gynecology, Women’s Hospital, Medicine School of Zhejiang University, Hangzhou, China

**Keywords:** cervical cancer, HPV16 E6, OCT4, NCOR1, p53, malignant progression

## Abstract

Human papillomaviruses (HPV), mainly HPV16 and HPV18, of high-risk classification are involved in cervical cancer carcinogenesis and progression. Octamer-binding transcription factor 4 (OCT4) is a key transcription factor that is increased in various cancer types. Cervical cancer patients with higher levels of OCT4 had worse survival rates. However, the definite mechanisms underlying its function in the development of cervical cancer still remain to be explicated. Here, our study demonstrated that OCT4 expression was slightly increased in cervical cancer tissues than in precancerous ones. However, OCT4 was significantly upregulated in HPV16-positive tissues, in contrast to the expression profiling for p53. Moreover, knockdown of HPV16 E6 in SiHa cells suppressed the expression of OCT4 with impaired activities of cell proliferation, migration, and invasion, while it recovered the expression of p53. Overexpression of OCT4 and p53 exerted opposite roles on cell proliferation, migration, invasion, and colony formation of cervical cancer cells. More importantly, the enforced expression of OCT4 augmented p53-inhibited cell migration, invasion, and colony formation in human cervical cancer by promoting EMT. Finally, we identified that OCT4 could bind to the *p53* promoter region to repress p53 expression by recruiting co-repressor NCOR1 using luciferase, ChIP, and co-IP experiments. We further illustrated that OCT4 not only increased the lung metastasis of cervical cancer but also effectively reversed p53-inhibited lung metastasis. In conclusion, our results suggested that HPV16 E6 activated the expression of OCT4 and subsequently crippled the transcription of p53 *via* co-repressor NCOR1, which contributed to cervical cancer progression.

## Introduction

Cervical cancer is the most common malignancy in women, and nearly 500,000 women every year are diagnosed with cervical cancer, resulting in more than 300,000 deaths worldwide (1). In China, the mortality and morbidity of cervical cancer remain very high (2). On many occasions, infection with high-risk types of human papillomavirus (HPV), that is HPV16 and HPV18, is considered the molecular epidemiologic evidence for cervical tumorigenesis. In recent decades, HPV vaccines are used to defend against high-risk HPV-related cancers 3, 4). However, the number of people inoculated with the vaccine remains very rare, especially in China. In addition, HPV vaccines cannot cure HPV-related diseases including cancer with advanced stages (4). For cervical cancer patients, the treatment options may still include surgery with chemotherapy and/or radiation therapy (5). However, the effectiveness of treatment is still not satisfactory for patients with malignant progression such as metastasis. Consequently, it is worth further demonstrating the molecular mechanism of cervical cancer progression for therapeutic target development.

It has been well documented that the HPV viral oncoprotein E6 could inactivate the tumor suppressor gene *TP53*, which functions as a transcription factor responding to an excess of cellular stresses including cell cycle regulators and immune tolerance factors (6–8). The activation of p53 is not merely of great importance in regulations for DNA repair, hypoxia, and apoptosis, but also for senescence, autophagy, and metabolism (9–12). p53 activation hinges on its intracellular stabilization, which is mediated by distinct post-translational modifications such as ubiquitination 13). Furthermore, in the course of embryonic stem cell differentiation, p53 directly represses the expression of certain genes, such as the pluripotency factor Nanog (14, 15). The transcription factor OCT4 takes a crucial role in maintaining self-renewal and pluripotency of embryonic stem cells (16). The OCT4 was related to cervical cancer susceptiveness, which colocalized in the nucleus with HPV16 E6, suggesting the potential role of viral HPV16 E6 in stem-like cancer cells through regulating OCT4 expression (17a). However, whether HPV16 E6-induced OCT4 expression could influence the level of p53 is still unknown.

In the present study, the expressions of p53 and OCT4 in cervical cancer tissues and cell lines under different HPV16 infection background were assessed, and the comprehensive functions of p53 and OCT4 overexpression on metastasis of cervical cancer were also investigated both *in vitro* and *in vivo*. We also further elucidated the underlying mechanism by which HPV16 E6-induced OCT4 repressed the expression of p53 at the transcriptional level. Our results provide distinctive comprehension into the mechanisms of progression in cervical cancer.

## Materials and Methods

### The Survival Prognosis Analysis

Kaplan–Meier Plotter (http://www.kmplot.com) is an online public database that evaluates the correlation between the expression of 30k genes and survival in 25k+ samples from 21 tumor types including lung, breast, gastric, cervical, or ovarian cancers. Here, we used the Kaplan–Meier Plotter dataset to analyze the prognostic significance of p53 or Oct4 mRNAs in cervical cancer using a log-rank test. Patients were split by auto select best cutoff and two survival types, namely, overall survival (OS) and recurrence-free survival (RFS) were included, respectively. No other restricted analysis was performed on the other subtypes, such as stage, gender, race, or grade. *p <*0.05 was considered statistically significant. The hazard ratios (HRs) with specific 95% confidence intervals (CIs) and *p*-values were listed.

### Cell Culture

The cervical cancer cell lines SiHa, HeLa, and C-33A were obtained from the American Type Culture Collection (Manassas, VA, USA). All cells were cultured in a humidified incubator with an atmosphere of 5% (v/v) CO_2_ at 37°C supplied with 100 μg/ml of streptomycin, 100 U/ml of penicillin, and 10% of FBS in Dulbecco’s modified Eagle’s medium (DMEM). All the reagents including DMEM, FBS, and antibiotics were purchased from Gibco Carlsbad, CA, USA.

### Clinical Specimen Collection and HPV Qualitative Detection

The sections of cervical cancer and their adjacent tissues were harvested at the Women’s Hospital, School of Medicine, Zhejiang University. Firstly, the Aptima HPV assay (Gen-Probe San Diego,CA, USA) was performed to detect the E6/E7 mRNA from 14 high-risk types of HPV in cervical specimens, and HPV E6/E7-negative and HPV E6/E7-positive tissues were distinguished. Then, the Aptima HPV16 18/45 genotype assay (Gen-Probe) was performed in the HPV E6/E7-positive CC tissues to differentiate HPV16 from HPV18 and HPV45. By this, we collected HPV-negative (14 high-risk types of HPV) and HPV-positive CC (HPV16 only) tissues for this study. All patients provided written informed consent for the use of these clinical materials in research, and the project was approved by the Institutional Ethics Committee of the hospital and was conducted in accordance with the Declaration of Helsinki.

### Immunohistochemistry

In brief, all sections were deparaffinized and rehydrated. Hydrogen peroxide (3%) in methanol was used to inhibit the activity of endogenous peroxidase. Heat-induced antigen retrieval (HIAR) was performed in all specimens with citrate buffer (0.01 M, pH 6.0) by using a steamer at 95°C. The primary antibodies were diluted into concentrations of 1:200 (OCT4) and 1:100 (p53), respectively, and incubated overnight at 4°C. Then, the samples were incubated with a Dako EnVision+ System-HRP Labelled Polymer for 30 min. Diaminobenzidine staining was then performed for 10 min. The samples were redyed with hematoxylin, dehydrated, coverslipped, and visualized. Immunohistochemical staining was assessed using semiquantitative scoring by two independent pathologists. The degree of staining was determined by the percentage of positive cells and the staining intensity.

### qPCR

By using TRIzol reagent (Invitrogen Carlsbad, CA, USA), the total RNA was extracted from the cervical cancer cell lines. By using the M-MLV RT Reagent Kit (Promega Madison, WI, USA), a reverse transcription product from 1 μg of total RNA was used to measure the expression of the indicated mRNAs. Quantitative real-time PCR (qPCR) was performed in triplicates on an Applied Biosystem 7300 quantitative PCR system (Applied Biosystems, Foster City, CA, USA) according to the manufacturer’s instructions. The PCR protocol included initial denaturation at 95°C for 10 min, followed by 40 cycles of denaturation at 95°C for 15 s and annealing and extension at 60°C for 1 min. The Ct values acquired from the different samples were contrasted using the 2^−ΔΔCt^ method. *β-Actin* was used as the internal reference gene. The sequences for the primers used are listed in **Table 1**.

**Table 1:** Primer sequences.

Name	F (5′–3′)	R (5′–3′)
OCT4	AGGGCGAAGCAGGAGTC	GATGGTCGTTTGGCTGAATA
p53	TGAGGTTGGCTCTGACTGTA	GTTTCTTCTTTGGCTGGGGA
HPV16 E6	TCAGGACCCACAGGAG	AACGGTTTGTTGTATTGC
β-Actin	AGCAGTTGTAGCTACCCGCCCA	GGCGGGCACGTTGAAGGTCT

### Western Blotting

Proteins were obtained from cervical cancer tissues and cells using RIPA lysis buffer including 1% of protease inhibitor cocktail (Sigma, St. Louis, MO, USA). After protein concentration determination using BCA assay, the samples were subjected to electrophoresis using 10%–12% of SDS–PAGE gel. Then, the proteins were shifted onto polyvinylidene difluoride membranes (PVDF membrane, Millipore, Darmstadt, Germany) followed by blocking with 5% fat-free milk for 2 h at room temperature (RT). Then, the membranes were incubated with primary antibodies against HPV16 E6 (Abcam ab70, Cambridge, UK; dilution rate: 1:500), OCT4 (Cell Signaling Technology, 2750S; dilution rate: 1:1,000), p53 (Proteintech 10442-1-Ap, Beverly MA; dilution rate: 1:500), N-cadherin (Proteintech Wuhan, China, 22018-1- Ap; dilution rate: 1:1,000), E-cadherin (Santa Cruz Biotechnology, sc-8426; dilution rate: 1:500), vimentin (Proteintech, 10336-1-Ap, Santa Cruz, CA, USA; dilution rate: 1:1,000), MMP7 (Proteintech, 10374-2-Ap, Wuhan, China; dilution rate: 1:1,000), MMP2 (Proteintech, 10373-2-Ap; dilution rate: 1:1,000), β-actin (Sigma, A5441; dilution rate: 1:5,000), or GAPDH (Cell Signaling Technology, 5174S; dilution rate: 1:5,000) overnight at 4°C. The corresponding horseradish peroxidase-conjugated secondary antibody was incubated for 1 h at room temperature. The signals were visualized after chemiluminescence reaction with horseradish peroxidase substrate.

### Transfection of Plasmid and siRNA

The overexpression plasmids for OCT4 and p53 were obtained from YouBao Bio (Changsha, China). The transfection was performed using Lipofectamine 2000 reagent according to the manufacturer’s instructions. Briefly, the plasmid was incubated with Lipofectamine 2000 (Invitrogen) for 20 min at RT to form a complex, and the transfection was executed for 24 h.

For gene silencing, siRNA against HPV16 E6 or NCOR1 was transfected with Lipofectamine 2000 reagent, and the protocol was similar to plasmid transfection. The sequence for siRNA against HPV16 E6 is 5′-GAGGUAUAUGACUUUGCUU-3′, the sequence against NOCR1 is 5′-GCAGUAUUGUCCAAAUUAUTT-3′, and the sequence against NC is 5′-UUCUCCGAACGU GUCACGUTT-3′ and 5′-ACGUGACACGUUCGGAGAATT-3′.

### Cell Viability Assay

Cell proliferation was analyzed in 96-well plates by the 3-(4,5-dimethylthiazol-2-yl)-2,5-diphenyltetrazolium bromide (MTT) experiment. In brief, 5,000 cervical cancer cells with indicated treatment were seeded into 96-well plates and incubated for different time periods. After incubation, the medium was discarded, and 50 μl of 1 mg/ml of MTT was added to each well and incubated at 37°C for 2–4 h. Then, 150 μl of DMSO was added to solubilize the purple formazan formed. Finally, a microplate reader (Molecular Devices Silicon Valley, United States) was used to read the absorbance at 570 nm.

### Migration and Invasion Assays

For the migration assay, the 8-μM Transwell system (Corning Corning, NY,USA) was used according to the manufacturer’s protocols. In brief, cells were seeded into the upper chambers after suspended in serum-free DMEM medium. The lower chambers were filled with 10% of FBS DMEM medium as a chemoattractant for a 24-h incubation. After that, the cells remaining in the upper chamber were eliminated. After fixation, the cells at the bottom of the insert were stained with crystal violet (0.5%) and counted under a microscope (Olympus Corp., Tokyo, Japan, CKX53). The results were calculated based on the average of three independent experiments. For the invasion assay, Matrigel was coated on the inserts (Corning) before cells were seeded, and the steps were the same as the migration assay.

### Colony Formation Assay

Briefly, 500 cells were seeded into a 6-well plate and incubated for 14 days. After incubation, the cells were fixed with methanol for 30 min at RT. After staining with crystal violet solution and rinsing with PBS for three times, the number of colonies was counted under a microscope (>50 cells).

### Generation of Stable Overexpression of OCT4 and/or p53 in HeLa Cells

To generate a stable overexpression of OCT4 and/or p53 in HeLa cells, the ZsGreen-control lentiviral particles and ZsGreen-OCT4 and/or P53 lentiviral particles were purchased from GenePharma (Shanghai, China). Then, the HeLa cells were infected with the concentrated virus with 5 μg/ml of Polybrene for 24 h. The infected HeLa cells were selected with 2 μg/ml of (sigma) puromycin to generate stable HeLa cells with overexpression of OCT4 or/and p53. The Western blot assay was used to confirm the expression of OCT4 and p53.

### Pulmonary Metastasis Model *In Vivo*

To execute the lung metastasis model of mice, 5 * 10^6^ stable overexpression of OCT4 and/or P53 HeLa cells was injected through the mice tail vein. After 21 days feeding, the mice were euthanized and sacrificed according to approved guidelines and on the basis of an approved protocol by the Zhejiang University Institutional Animal Care and Use Committee. The lungs were removed integrally and photographed. The tissues were also stained with hematoxylin and eosin (HE) to assess the HeLa cell metastasis in the lungs.

### Luciferase Reporter Assay

In brief, the promoter fragments of the *p53* gene comprising the potential OCT4 binding site or with the mutant binding site were inserted into PGL3 vectors (Promega). The SiHa cells were co-transfected with PGL3 reporter plasmids with wild-type p53 promoter or mutant p53 promoter in combination with pcDNA-OCT4 alone or with NCOR1-siRNA for 24 h. The cells were harvested and lysed. The activity of luciferase was measured by a Luciferase reporter assay kit (Promega), and pCMV-Renilla reporter plasmid was used as a normalizer.

### Co-Immunoprecipitation

The SiHa cells were lysed in immunoprecipitated lyse−bind−wash buffer [50 mM of Tris–HCl, 150 mM of NaCl, 1% of NP−40 (v/v), 2 mM of EDTA] including 1% of protease inhibitor cocktail (Sigma) for 10 min on ice. Then, the samples were centrifuged at 14,000×*g* for 10 min at 4°C. After that, the samples (800 μg protein) were incubated with Flag magnetic beads (Sigma) overnight at 4°C. The IP complex was obtained using a magnetic lock and rinsed with PBS. Then, the samples were subjected to Western blotting analysis by using anti−Flag, OCT4, or NCOR1 antibodies.

### Chromatin Immunoprecipitation Assay

Chromatin immunoprecipitation (ChIP) assays were fulfilled using anti-OCT4 or anti-NCOR1 antibody (Cell Signaling Technology) and protein A-agarose beads (Millipore) as described according to the ChIP kit (Cell Signaling Technology) protocols. The p53 promoter DNA in the ChIP product was measured by qPCR using gene-specific primers. The PCR primer sequences are 5′-GGGTGAGTGGGATGGAAG-3′ (forward) and 5′-CGGGTGGATGTGCAA AGA-3′ (reverse).

### Statistical Analysis

The data were presented as the means ± SD from three independent experiments. Statistical significance, in which *p <*0.05 (*) was considered significant, was decided by using the two-sided Student’s *t*-test for two groups or the one-way ANOVA for multiple groups. The correlation between the immunohistochemistry (IHC) results of p53 and OCT4 proteins and the clinicopathological features was evaluated by the non-parametric tests. The GraphPad Prism 5.0 (GraphPad Software, Inc., La Jolla, CA, USA) and SPSS 13.0 (SPSS, Inc., Chicago, IL, USA) software packages were used to perform all statistical analyses.

## Results

### OCT4 and p53 Expression and Their Clinical Significance in Cervical Cancer

**Figure 1:**
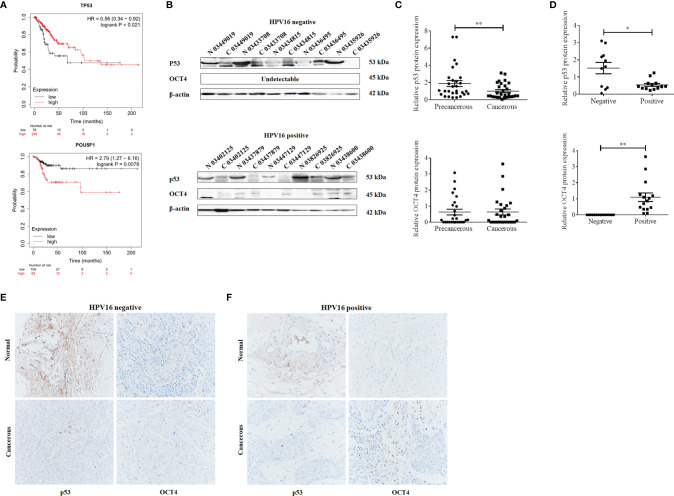
Octamer-binding transcription factor 4 (OCT4) and p53 expression and their clinical significance in cervical cancer. **(A)** The association between the expression level of OCT4 or p53 and the overall survival rate of cervical cancer patients. Data were obtained from the Kaplan–Meier Plotter database. **(B)** Western blot analysis of OCT4 and p53 expression in human cervical cancer tissues and precancerous ones. **(C, D)** Statistical analysis of OCT4 and p53 protein expression in HPV16-negative or HPV16-positive human cervical cancer tissues by the WB assay. **(E, F)** Graphs showing OCT4 and p53 expression in HPV16-negative or HPV16-positive human cervical cancer tissues detected by immunohistochemistry. Brown color displays OCT4 or p53 proteins, with counterstaining by hematoxylin in blue. *p < 0.05, **p < 0.01 vs. control.

To analyze the clinical relevance of OCT4 and p53 mRNA levels in human cervical cancer, the database Kaplan–Meier Plotter was used and the results indicated that a high level of OCT4 mRNA was significantly negatively associated with the RFS rate of cervical cancer patients (*p* = 0.0078), while a high level of p53 mRNA was significantly positively related with the OS rate of cervical cancer patients (*p* = 0.021) (**Figure 1A**). However, no significant statistical difference was obtained in the associations between OCT4 mRNA levels and OS (*p* = 0.25) and between p53 mRNA levels and RFS (*p* = 0.14) in cervical cancer (data not shown). We also examined the protein expression levels of OCT4 and p53 in cervical cancer specimens by Western blot and IHC assays. The representative figures of the Western blot (WB) data are shown in **Figure 1B**. Our data revealed that the p53 protein was significantly lower expressed in cervical cancer tissues than in precancerous ones, while no obvious difference in OCT4 protein levels was observed between the two groups, and in most tissues, the OCT4 protein was undetectable (**Figure 1C**). Next, the expression profiling of both proteins in cervical cancer tissues with different HPV16 infection background was analyzed. As shown in **Figure 1D**, the p53 protein was significantly reduced in HPV16-positive cancer tissues, in which the OCT4 protein was significantly increased. More interestingly, in almost all HPV16-negative cancer tissues, the OCT4 protein was not detected (**Figure 1D**). The IHC assay was also used to assess the OCT4 and p53 protein expression levels in clinical samples. A similar expression profiling of OCT4 and p53 was observed in the IHC data, and the representative pictures are shown in **Figures 1E, F**. Finally, statistical analysis of IHC staining results indicated that the expression level of p53 (**Table 2**) was negatively associated with patient stages (*p* = 0.001) and HPV16 (*p* = 0.047), while the expression level of OCT4 (**Table 3**) was positively associated with patient stages (*p* = 0.026), lymph node metastasis (*p* = 0.025), and HPV16 E6 (*p* = 0.006), respectively. However, age or histological type was not associated with the expression of both proteins.

**Table 2:** Association between p53 protein expression and clinicopathological features in cervical cancer patients.

Character	Level	Low expression of p53	High expression of p53	*p*-value
Age	≤60	45.0%	55.0%	0.176
	>60	83.3%	16.7%	
Histological type	G1	50%	50%	0.263
	G2	43.8%	56.3%	
	G3	83.3%	16.7%	
	G4	–	–	
Stage	I	10.0%	90.0%	0.001^**^
	II	72.7%	27.3%	
	III	100%	0%	
	IV	–	–	
Lymph node metastasis	+	66.7%	53.8%	0.426
	−	47.1%	52.9%	
HPV16 E6	+	73.3%	26.7%	0.047^*^
	−	27.3%	72.7%	

*p < 0.05; **p < 0.01.

**Table 3:** Association between OCT4 protein expression and clinicopathological features in cervical cancer patients.

Character	Level	Low expression of OCT4	High expression of OCT4	*p*-value
Age	≤60	50%	50%	0.573
	>60	33.3%	66.7%	
Histological type	G1	50%	50%	0.263
	G2	62.5%	37.5%	
	G3	16.7%	83.3%	
	G4	–	–	
Stage	I	90%	10%	0.026^*^
	II	18.2%	81.8%	
	III	40%	60%	
	IV	–	–	
Lymph node metastasis	+	57.1%	42.9%	0.025^*^
	−	47.4%	52.6%	
HPV16 E6	+	33.3%	66.7%	0.006^**^
	−	72.7%	27.3%	

*p < 0.05; **p < 0.01.

### HPV16 E6 Promoted Cell Proliferation, Migration, and Invasion in Human Cervical Cancer Cells by Upregulated OCT4 Expression

**Figure 2:**
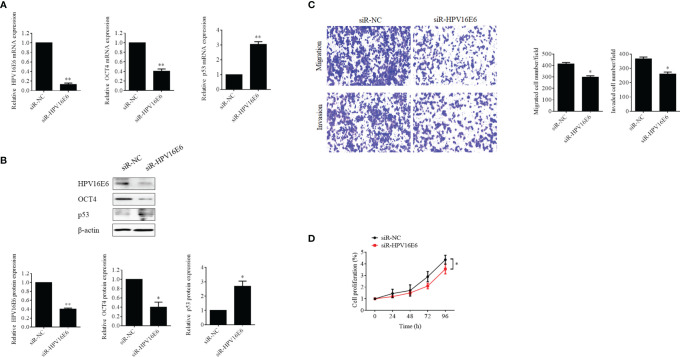
Silencing of HPV16 E6 repressed OCT4 expression, cell proliferation, migration, and invasion and stimulated p53 expression in human cervical cancer cells. **(A)** The SiHa cells were transfected with HPV16 E6-siRNA or control siRNA, for 24 h. Then, HPV16 E6, OCT4, and p53 mRNA levels were determined by RT-qPCR. **(B)** Then, E6, OCT4, and p53 protein levels were detected by Western blot in cells treated as in **(A)**. **(C)** Transwell assay analysis of migration and invasion abilities in the human cervical cancer cell line SiHa transfected with HPV16 E6-siRNA or control siRNA. **(D)** MTT assay analysis of proliferation ability in the human cervical cancer cell line SiHa transfected with HPV16 E6-siRNA or control siRNA. *p < 0.05, **p < 0.01 vs. control.

To examine the role of HPV16 E6 in the expression of OCT4 and p53 in cervical cancer, the HPV16 E6-positive SiHa cells were used. As illustrated in **Figures 2A, B**, HPV16 E6 silencing not only effectively decreased the mRNA and protein levels of OCT4 but also increased those of p53 in SiHa cells. Moreover, the data from the Transwell assay indicated that HPV16 E6 silencing significantly reduced the potential of cell migration and invasion in SiHa cells (**Figure 2C**). Lastly, our data also confirmed that knockdown of HPV16 E6 obviously lessened SiHa cell growth activity compared to the siRNA control groups (**Figure 2D**).

**Figure 3:**
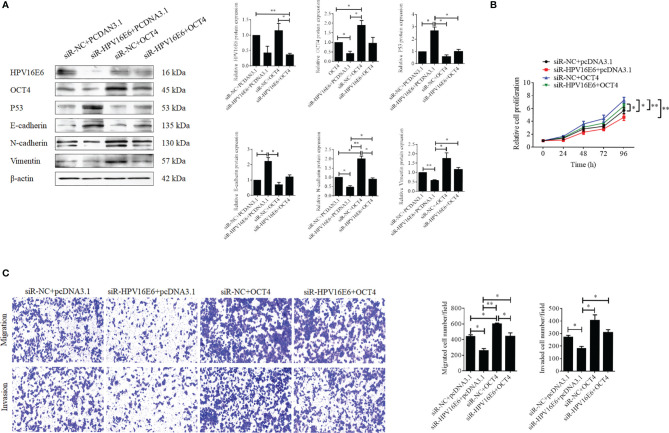
OCT4 overexpression blocked HPV16 E6 silencing inhibiting cell proliferation, migration, invasion, and EMT in human cervical cancer cells. **(A)** The SiHa cells were co-transfected with HPV16 E6-siRNA and/or pcDNA-OCT4 for 24 h. Then, the protein levels of HPV16 E6, OCT4, p53, E-cadherin, N-cadherin, and vimentin were detected by the Western blot assay. **(B)** MTT assay analysis of proliferation ability in SiHa cells treated as in **(A)**. **(C)** Transwell assay analysis of migration and invasion abilities in SiHa treated as in **(A)**. *p < 0.05, **p < 0.01 vs. control.

To verify the role of OCT4 in the biofunctions of HPV16 E6 in cervical cancer cells, SiHa cells were transfected with HPV16 E6-siRNA or pcDNA-OCT4 alone or in combination. Firstly, HPV16 E6 silencing impaired the epithelial–mesenchymal transition (EMT) in SiHa cells by enhancing the expression of E-cadherin and reducing the expression of vimentin and N-cadherin (**Figure 3A**). However, overexpression of OCT4 reduced the upregulated expression of E-cadherin, while it increased the downregulated expression of N-cadherin, which was mediated by HPV16 E6 silencing in SiHa cells (**Figure 3A**). Furthermore, overexpression of OCT4 effectively recovered the lessened cell proliferation in SiHa cells transfected with HPV16 E6 siRNA (**Figure 3B**). Finally, the roles of OCT4 in cell migration and invasion in SiHa cells with HPV16 E6 silencing were also elucidated. As shown in **Figure 3C**, the ectopic expression of OCT4 not only significantly elevated the cell migration and invasion activities in SiHa cells but also obviously regained those potentials which were weakened by HPV16 E6 silencing. Therefore, these data demonstrated that the ectopic expression of OCT4 could effectively regain the cell proliferation, migration, invasion, and EMT in cervical cancer cells inhibited by the knockdown of HPV16 E6.

### OCT4 and p53 Exert Opposite Roles in the Regulation of Malignant Phenotypes in Cervical Cancer Cells

**Figure 4:**
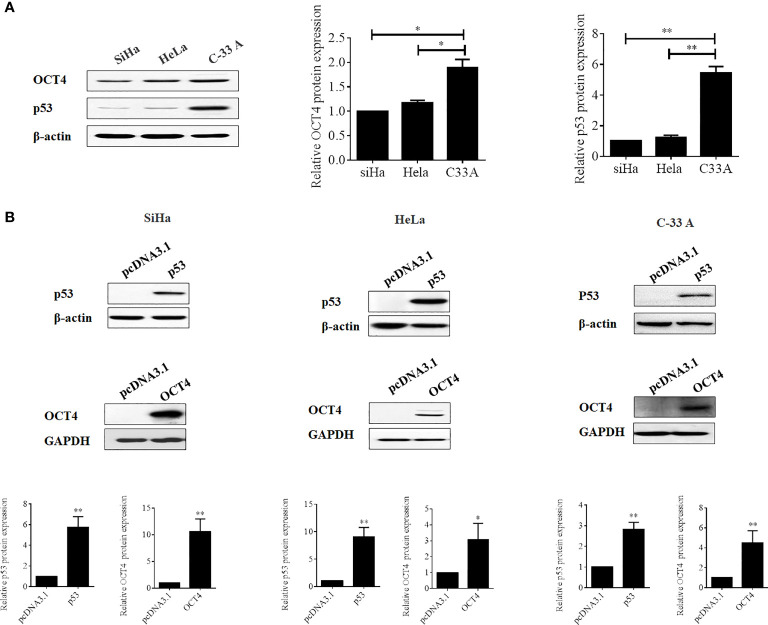
Evaluation of the efficacies of OCT4 and p53 overexpression plasmids in cervical cancer cells. **(A)** Western blot analysis of the basal protein levels of OCT4 and p53 in SiHa, HeLa, and C-33A cells; β-actin was used as a control. **(B)** Western blot analysis of the efficacies of OCT4 and p53 overexpression plasmids transfected in SiHa, HeLa, and C-33A cells; GAPDH and β-actin were used as controls. *p < 0.05, **p < 0.01 vs. control.

**Figure 5:**
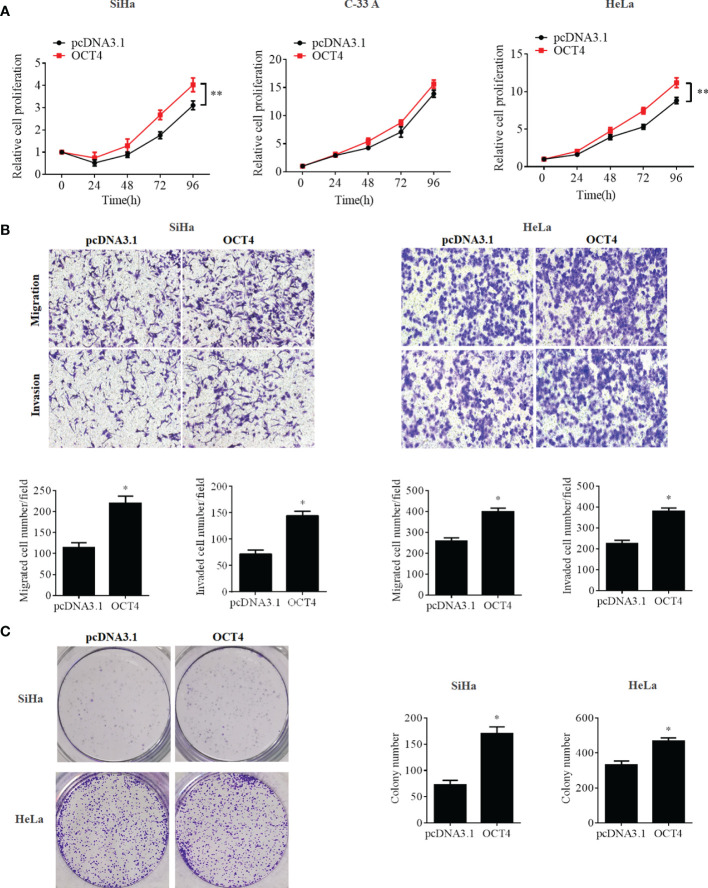
OCT4 overexpression promoted cell proliferation, migration, invasion, and colony formation activities in human cervical cancer cells. **(A)** MTT assay analysis of proliferation abilities in the human cervical cancer cell lines SiHa, C-33A, and HeLa transfected with pcDNA3.1-OCT4 or pcDNA3.1. **(B)** Transwell assay analysis of migration and invasion abilities in the human cervical cancer cell lines SiHa and HeLa transfected with pcDNA3.1-OCT4 or pcDNA3.1. **(C)** Representative photographs of OCT4 overexpression on colony formation in SiHa and HeLa cells. *p < 0.05, **p < 0.01 vs. control.

**Figure 6:**
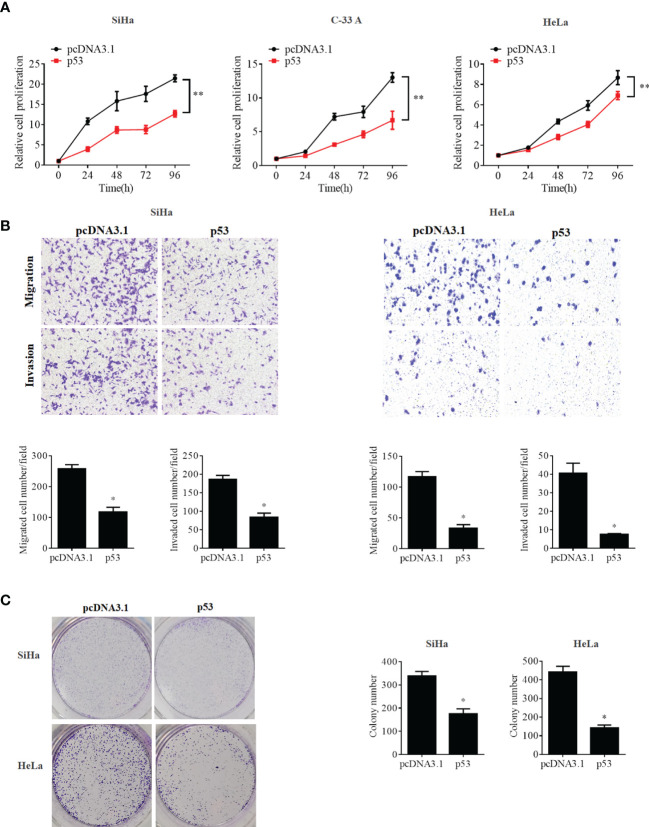
p53 overexpression inhibited cell proliferation, migration, invasion, and colony formation potentials in human cervical cancer cells. **(A)** MTT assay analysis of proliferation ability in the human cervical cancer cell lines SiHa, C-33A, and HeLa transfected with pcDNA3.1-p53 or pcDNA3.1. **(B)** Transwell assay analysis of migration and invasion abilities in the human cervical cancer cell lines SiHa and HeLa transfected with pcDNA3.1-p53 or pcDNA3.1. **(C)** Representative photographs of p53 overexpression on colony formation in SiHa and HeLa cells. *p < 0.05, **p < 0.01 vs. control.

To further confirm whether the OCT4/p53 axis played indispensable functions in the progression of cervical cancer, the background expression of these two proteins in human cervical cancer cell lines was examined. Our data indicated that the expression of the p53 protein in HPV-negative C-33A cells was much higher than those in HPV-positive SiHa and HeLa cells (**Figure 4A**). This result could mainly be due to the HPV-negative background of C-33A cells, as one of the most important mechanisms for HPV16 E6 contributing to the development and progression of cervical cancer is to promote the degradation of the p53 protein (18). To our surprise, the highest level of OCT4 was also observed in C-33A cells (**Figure 4A**). Firstly, OCT4 and p53 overexpression plasmids were used to transfect the three cell lines and the expression efficiency was also confirmed by the WB assay. As shown in **Figure 4B**, both OCT4 and p53 proteins were effectively overexpressed in three cell lines. Next, the MTT assay was chosen to determine the roles of OCT4 and p53 overexpression in cervical cancer cell proliferation. As shown in **Figures 5A**, **6A**, OCT4 could effectively promote the cell proliferation potentials (**Figure 5A**), while p53 could obviously inhibit those in all three cell lines (**Figure 6A**). Similar results were also obtained in the Transwell assays. Our data revealed that OCT4 overexpression significantly upregulated the cell migration and invasion potentials in SiHa and HeLa cells (**Figure 5B**), while the ectopic expression of p53 exerted opposite functions in the same cell lines (**Figure 6B**). Finally, colony formation assays were performed and the data demonstrated that OCT4 slightly enhanced the colony formation activity (**Figure 5C**), but p53 dramatically suppressed those in SiHa and HeLa cells (**Figure 6C**).

### The Role of the OCT4/p53 Axis in Malignant Progression of Cervical Cancer Cells by Promoting EMT and MMP Expression

**Figure 7:**
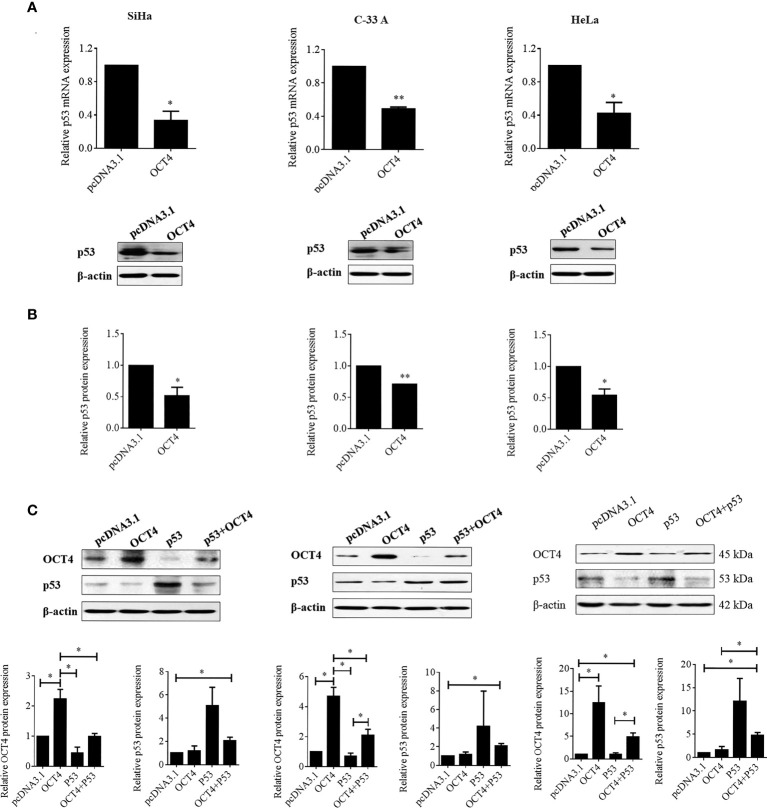
OCT4 overexpression inactivated p53 expression in human cervical cancer cells. **(A)** The SiHa, C-33A, and HeLa cells were transfected with pcDNA3.1-OCT4 or pcDNA3.1 for 24 h. Then, p53 mRNA levels were determined by RT-PCR. **(B)** p53 protein levels were detected by Western blot in cells treated as in **(A)**. **(C)** The SiHa, C-33A, and HeLa cells were co-transfected with pcDNA3.1-OCT4 or pcDNA3.1-p53 or the empty vector pcDNA3.1 for 24 h. Then, the OCT4 and p53 protein levels were detected by Western blot. *p < 0.05, **p < 0.01 vs. control.

**Figure 8:**
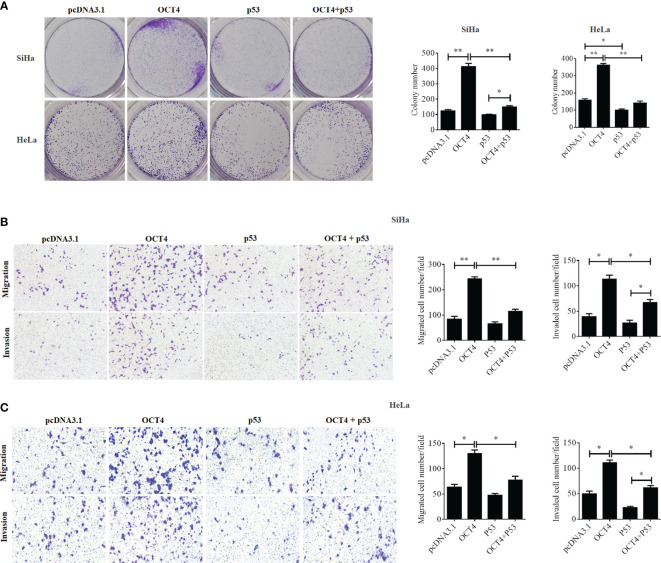
OCT4 and p53 exerted opposite functions on cell migration, invasion, and colony formation activities in human cervical cancer cells. **(A)** The SiHa and HeLa cells were transfected with pcDNA3.1-OCT4 or pcDNA3.1-p53 alone or in combination. After transfection, representative photographs of colony formation in SiHa and HeLa cells were shown. **(B)** Transwell assay analysis of migration and invasion abilities in SiHa cells treated as in **(A)**. **(C)** Transwell assay analysis of migration and invasion abilities in HeLa cells treated as in **(A)**. *p < 0.05, **p < 0.01 vs. control.

Although a recent study has reported that p53 could influence the expression of OCT4, no research was focused on whether OCT4 could directly regulate p53 expression in cervical cancer (19). In the present study, the p53 mRNA and protein expression levels were examined in SiHa, HeLa, and C-33A cells with OCT4 overexpressed. As shown in **Figures 7A, B** the ectopic expression of OCT4 significantly downregulated both the mRNA and protein expression levels of p53 in all three cell lines. Furthermore, all three cell lines were transfected with OCT4 or p53 overexpression plasmid alone or in combination, and their protein expression levels were analyzed by the WB assay. The enhanced expression of p53 indeed downregulated the OCT4 expression which was consistent with the published study, while OCT4 overexpression decreased the expression of the p53 proteins in cells and even co-transfected the p53 overexpression plasmid (**Figure 7C**). In fact, we also analyzed the potential binding sites of the cmv promoter sequence in the pcDNA3.1 vector by using the PROMO dataset, and the potential binding sites for both p53 and OCT4 were found on the promoter sequence (data not shown). This finding could also explain appropriately why the co-expression of both could affect the levels of both proteins. Subsequently, HeLa and SiHa cells were chosen to determine the roles of the OCT4/p53 axis on malignant phenotypes of cervical cancer cells. Several lines of evidence have supported that OCT4, functioning as an oncogene, promoted the malignant progression of cervical cancer by upregulating the colony formation, cell migration, and invasion activities (20, 21). As shown in **Figure 8**, OCT4 significantly augmented the activities of colony formation, cell migration, and invasion in two cell lines, which were slightly weakened by p53. More importantly, the ectopic expression of OCT4 effectively improved these phenotypes which were suppressed by p53 (**Figure 8**).

**Figure 9:**
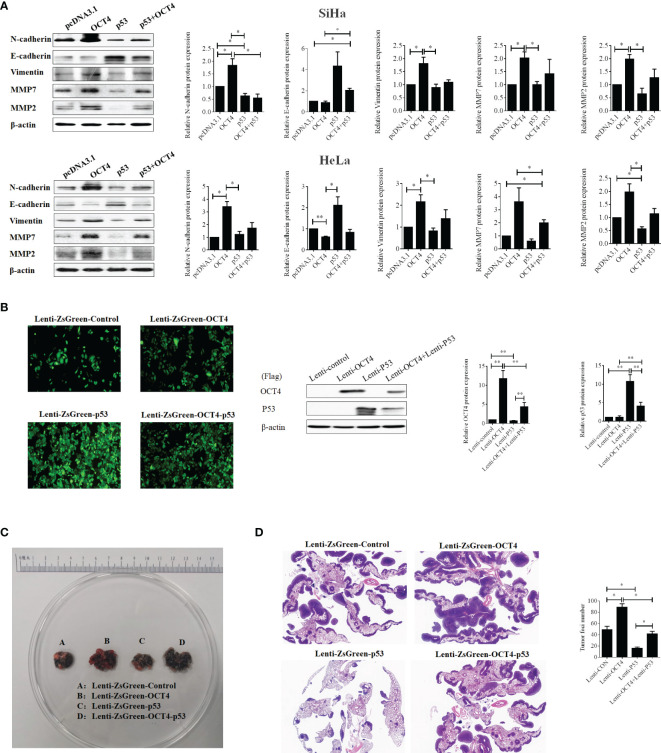
The ectopic expression of OCT4 regained the metastasis of cervical cancer inhibited by p53 *via* promoting the EMT and the expression of MMP7 and MMP2. **(A)** The SiHa and HeLa cells were transfected with pcDNA3.1-OCT4 and pcDNA3.1-p53 alone or in combination, and the protein was extracted after 24 h of transfection. Then, the protein levels of N-cadherin, E-cadherin, vimentin, MMP7, and MMP2 were detected by Western blot. **(B)** Representative photographs for HeLa cells with stable overexpression of OCT4 and/or p53 examined by a fluorescence microscope (left panel), while the WB assay was used to determine the expression of OCT4 and p53 proteins (right panel). **(C)** Lung images of nude mice injected with HeLa cells with stable overexpression of OCT4 and/or p53 *via* the tail vein. **(D)** HE staining of lung images of nude mice injected with HeLa cells with stable overexpression of OCT4 and/or p53 *via* the tail vein. *p < 0.05, **p < 0.01 vs. control.

Then, we found that p53 overexpression remarkably upregulated the epithelial marker E-cadherin and reduced the expression levels of mesenchymal markers N-cadherin and MMP2 in SiHa cells, while it upregulated the expression of E-cadherin and reduced the expression levels of vimentin, MMP2, and MMP7 in HeLa cells. However, co-overexpression of OCT4 and p53 distinctly increased the expression levels of N-cadherin, vimentin, MMP7, and MMP2 and extremely decreased E-cadherin compared to the only p53 overexpression groups (**Figure 9A**). Therefore, these data also authenticated that the combined overexpression of OCT4 and p53 significantly showed the reversal results only in p53 overexpression groups.

### The Ectopic Expression of OCT4 Regained Cervical Cancer Lung Metastasis Impaired by p53

To prove the above results *in vivo*, SiHa cell lines with stable overexpression of OCT4 or p53 alone or in combination were constructed to perform the lung metastasis experiments in nude mice. Initially, the samples emitting a bright green light viewed by a fluorescence microscope certified that stable expression was successfully constructed (**Figure 9B**). Then, as shown in **Figure 9C**, OCT4 overexpression heightened the number of metastatic nodules in the lungs, while p53 overexpression abated the number of nodules. Meanwhile, co-overexpression of OCT4 and p53 promoted the SiHa cell metastasis compared with the p53 overexpression group. Similar results were obtained from the data of HE staining (**Figure 9D**). Taken together, these data certified that OCT4 strengthened p53-repressed lung metastasis of cervical cancer *in vivo*.

### OCT4 Suppressed p53 Expression in Cervical Cancer *via* Co-Repressor NCOR1

**Figure 10:**
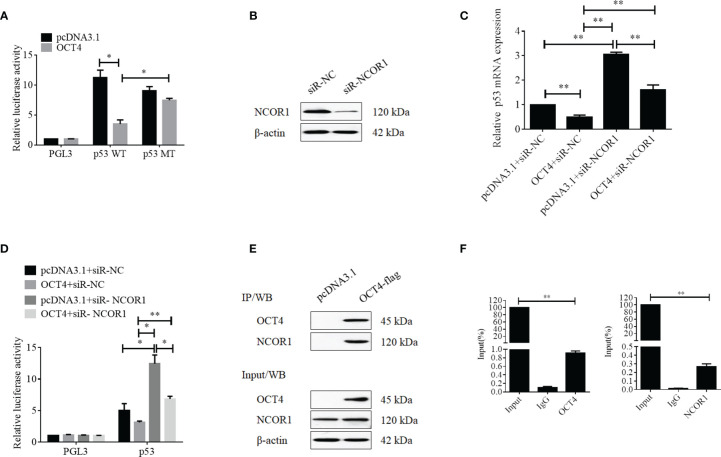
OCT4 suppressed p53 expression by binding to the promoter with co-repressor NCOR1. **(A)** Relative luciferase activity of SiHa cells transfected with the PGL3, p53-wild type, or p53-mutated constructions was measured under normal or overexpression of OCT4. **(B)** The SiHa cells were transfected with control-siRNA or NCOR1-siRNA for 24 h. Then, the NCOR1 protein levels were detected by Western blot. **(C)** The SiHa cells were co-transfected with pcDNA3.1-OCT4 and/or NCOR1-siRNA for 24 h. Then, p53 mRNA levels were determined by RT-qPCR. **(D)** Relative luciferase activity of PGL3-p53 promoter in SiHa cells transfected with OCT4 overexpression plasmid and/or NCOR1-siRNA for 24 h. **(E)** The interaction between OCT4 and NCOR1 was verified by co-IP and Western blotting assays. **(F)** The ChIP/qPCR assay was performed to analyze the binding potential of OCT4 and NCOR1 in the p53 promoter region. *p < 0.05, **p < 0.01 vs. control.

To further discover the underlying mechanisms by which OCT4 repressed the expression of p53, luciferase reporter assay was performed. Firstly, we analyzed the p53 promoter sequence using the online transcriptional factor binding site prediction software PROMO and found that OCT4 could bind to the p53 promoter fragment of +278 to +288 (5′-AGTTGCATTGT-3′). Therefore, the core p53 promoter sequences that contained the wild-type or mutant potential binding sites were synthesized and subcloned to the PGL3 vector to construct the luciferase reporter plasmids. Dual reporter luciferase assay was performed, and the data indicated that OCT4 could effectively repress the luciferase activity of the wild-type p53 promoter but failed in the mutant one (**Figure 10A**). As a well-known co-repressor for OCT4, we also tried to confirm whether NCOR1 was involved in the suppression of p53 mediated by OCT4 in cervical cancer cells in this study (22). We firstly identified the silencing efficiency of siRNA against NCOR1, and as shown in **Figure 10B**, NCOR1 protein expression was effectively downregulated after transfection with siRNA. More importantly, the subsequent data revealed that silencing of NCOR1 significantly recovered the mRNA expression of p53 impaired by OCT4 (**Figure 10C**). Consistently, luciferase assay results also supported that NCOR1 silencing could effectively reverse the role of OCT4 on the p53 promoter (**Figure 10D**). Subsequently, the co-immunoprecipitation (co-IP) experiment was used to verify whether OCT4 and NCOR1 interacted with each other. As shown in **Figure 10E**, co-IP data also confirmed that OCT4 indeed interacted with NCOR1 in the cervical cancer cell line SiHa. Finally, the ChIP assay was performed using OCT4 or NCOR1 antibodies, and the IP products were analyzed using qPCR to detect the binding of the p53 promoter fragments with the primers across the potential binding site of OCT4. As shown in **Figure 10F**, both OCT4 and NCOR1 could enrich the p53 promoter. Taken together, all these findings suggested that these two proteins could bind to the p53 promoter *via* the site (5′-AGTTGCATTGT-3′) and inhibited the p53 expression.

## Discussion

It has been well acknowledged that the HPV16 E6 oncoprotein plays crucial roles in the process of carcinogenesis and maintenance of malignant phenotypes in various cancers, especially in cervical cancer (18, 23–25). This is primarily due to HPV16 E6-induced posttranslational modification of p53 and promotes its degradation by 26S proteasome (18). In the present study, we found another regulation pathway, by which HPV16 E6 repressed the transcription of p53 *via* activating the OCT4 expression *via* recruiting the co-repressor NCOR1. OCT4 expression has been proven to be related to an undifferentiation phenotype of cancers and poor prognosis in tumor patients (26). OCT4 plays an important role in numerous biological processes such as epigenetic regulation, chromatin remodeling, and transcriptional modulation in embryonic stem cells (27). For instance, OCT4 collaborated with NCOR1 precipitated transcriptional repression on high expression genes in MEFs (22). Additionally, previous studies suggested that HPV16 E6 upregulated OCT4 expression by targeting HDAC1 for proteasomal degradation (17, 28b). Moreover, JNK-phosphorylated OCT4 reduced OCT4 protein stability which was enhanced by KAP1 overexpression (29, 30).

In the current study, we found that p53 was lower expressed in cervical cancer tissues than in precancerous ones, but no statistically significant difference was observed in OCT4. To further confirm the relationship of OCT4 and p53 expression profiling with HPV16 background in cervical cancer, the Aptima HPV assay was used for the selection of HPV-negative tissues, while the Aptima HPV16 18/45 genotype assay was performed to obtain the HPV16-positive tissues for the subsequent experiments. Interestingly, our data indicated that OCT4 was higher expressed in HPV16-positive than in HPV16-negative cervical cancer tissues, while the expression profiling for p53 was the opposite. Meanwhile, there was a significantly negative relevance between OCT4 expression level and RFS rate of cervical cancer patients. Furthermore, HPV16 E6 silencing resulted in the downregulation of OCT4 expression, cell proliferation, migration, invasion, and EMT, while it activated the p53 expression in cervical cancer cells. Interestingly, OCT4 overexpression accelerated cell proliferation and metastasis and inactivated p53, whose overexpression repressed cell proliferation, metastasis, and colony formation in cervical cancer. Although it is well known that p53 is a positive regulator for apoptosis, our data indicated no obvious increase in apoptosis in SiHa and HeLa cells after transfected with the p53 overexpression plasmid (data now shown). These findings also excluded the possibility that suppression in cell proliferation, migration, and invasion is a side effect of apoptosis induced by p53 overexpression. Moreover, co-overexpression of OCT4 and p53 significantly enhanced p53 overexpression blocking cell metastasis. Using a lung metastasis model in nude mouse, we illustrated that OCT4 heightened p53-suppressed lung metastasis *in vivo.*

Mechanistically, we also elucidated how OCT4 regulated the transcription of *p53* in cervical cancer in this study. By analyzing the potential binding site of OCT4 on the p53 promoter by the online predictor software, a potential binding site was found. By constructing the luciferase reporter plasmids with wild-type or mutant p53 promoters, we initially confirmed that OCT4 could weaken the luciferase activity of the p53 promoter *via* the potential binding site. As we have known, a co-repressor is indispensable when OCT4 acts as a transcriptional repressor. Here, we identified the role of NCOR1, a co-repressor for OCT4, in the transcriptional regulation of p53 mediated by OCT4. Our data revealed that silencing of NCOR1 using siRNA could not only regain the mRNA expression of p53 impaired by OCT4 but also recover the suppressed luciferase activity of the p53 promoter. Finally, and most importantly, the data of the ChIP analysis proved that OCT4 cooperated with NCOR1 bound to the p53 promoter region to inactive p53 expression.

In conclusion, our findings suggested that HPV16 E6-activated OCT4 inhibited p53 expression by collaborating with NCOR1 and facilitated cell proliferation and metastasis in human cervical cancer. Our findings provide a novel insight into how the HPV16 E6/OCT4/p53 axis contributes to the development and progression of human cervical cancer.

## Data Availability Statement

The raw data supporting the conclusions of this article will be made available by the authors, without undue reservation.

## Ethics Statement

The studies involving human participants were reviewed and approved by the Institutional Ethics Committee of the Women’s Hospital, Medicine School of Zhejiang University (Hangzhou, China). The patients/participants provided their written informed consent to participate in this study. The animal study was reviewed and approved by the Institutional Ethics Committee of the Women’s Hospital, Medicine School of Zhejiang University (Hangzhou, China).

## Author Contributions

WL and PJ conceived and designed the experiments. SS, ZL, LL, XY, YZ, TW, and XZ performed the experiments. PJ analyzed the data and wrote the manuscript. All authors contributed to the article and approved the submitted version.

## Funding

This work was supported by the National Natural Science Foundation of China (grant number 81902625) and Zhejiang Provincial Natural Science Foundation of China under Grant No. LQ19H160028.

## Conflict of Interest

The authors declare that the research was conducted in the absence of any commercial or financial relationships that could be construed as a potential conflict of interest.

## Publisher’s Note

All claims expressed in this article are solely those of the authors and do not necessarily represent those of their affiliated organizations, or those of the publisher, the editors and the reviewers. Any product that may be evaluated in this article, or claim that may be made by its manufacturer, is not guaranteed or endorsed by the publisher.
